# Epidemiological Characteristics and Risk Factors of Methamphetamine—Associated Psychotic Symptoms

**DOI:** 10.3389/fpsyt.2018.00489

**Published:** 2018-10-12

**Authors:** Meng-Fan Su, Mo-Xuan Liu, Jin-Qiao Li, Julia M. Lappin, Su-xia Li, Ping Wu, Zhi-Min Liu, Jie Shi, Lin Lu, Yanping Bao

**Affiliations:** ^1^National Institute on Drug Dependence and Beijing Key Laboratory of Drug Dependence, Peking University, Beijing, China; ^2^School of Public Health, Peking University, Beijing, China; ^3^National Drug and Alcohol Research Centre, University of New South Wales, Sydney, NSW, Australia; ^4^Key Laboratory of Mental Health and Peking University Sixth Hospital, National Clinical Research Center for Mental Disorders, Institute of Mental Health, Peking University, Beijing, China; ^5^Peking-Tsinghua Center for Life Sciences and IDG/McGovern Institute for Brain Research, Peking University, Beijing, China

**Keywords:** MA-associated psychotic symptoms, risk factors, prevalence, MA users, China

## Abstract

**Objective:** To explore the epidemiological characteristics and the risk factors for methamphetamine (MA)—associated psychotic symptoms among MA users in China.

**Methods:** A cross-sectional study was conducted between April, 2012 and October, 2015 among individuals for whom MA was the principal drug of use in a Compulsory Drug Detoxification Center in Beijing, Guangdong Province. Demographic, drug use and psychological characteristics were examined using a specifically-designed questionnaire, the Positive and Negative Syndrome Scale, Barratt Impulsive Scale, Hamilton Anxiety Scale and Beck Depression Inventory. Logistic regression was performed to explore the risk factors for MA-associated psychotic symptoms.

**Results:** A total of 1685 participants were included. Participants were predominantly aged 30 or above, unemployed, and were unmarried Han Chinese men, with limited education. The duration of MA use was more than 3 months in 72.3%. 47.8% reported that the dose of MA use was ≥ 0.2 g per occasion of use. 11.5% had used two or more synthetic drugs. The prevalence of MA-associated psychotic symptoms was 17.0% among MA users during periods of abstinence. Multiple logistic regression analyses showed that a higher dose (≥0.2 g per time), a longer duration of MA use (>3 months) a history of heroin use and a history of tobacco use were associated with MA-associated psychotic symptoms, with adjusted odds ratios (ORs) of 1.96 (95% confidence interval [CI]: 1.40–2.76), 1.98 (95% CI: 1.33–2.96) and 2.45 (95% CI: 1.67–3.60), 1.78 (95% CI: 1.27–2.49) respectively. MA-associated psychotic symptoms were less common among married/cohabitating than unmarried (OR = 0.56; 95% CI: 0.39–0.81), and unemployed than employed (OR = 0.65; 95% CI: 0.47–0.92) individuals. MA users with anxiety and depression symptoms had significantly greater risk for MA-associated psychotic symptoms by 9.70 (6.92–13.59) and 1.90 (1.36–2.65) times respectively. Individuals with higher impulsivity were more likely to have MA-associated psychotic symptoms than those with lower (OR = 2.19; CI:1.50–3.20).

**Conclusion:** MA-associated psychotic symptoms occurred frequently among MA users in China. The efforts that facilitate drug users' attempts to reduce MA use, abstain from poly-drug use, and control associated psychiatric symptoms and impulsivity should be supported because of their potential contribution to MA-associated psychotic symptoms in this population.

## Introduction

Amphetamine-type stimulants (ATS) comprising predominantly methamphetamine (MA) are the second most commonly used illicit drug globally after cannabis. Their use is associated with health problems globally, and particularly in East and South-East Asia (1, 2). The United Nations Office on Drugs and Crime reported that an estimated 37 million individuals have used amphetamines in the past year and that MA was the most consumed drug in China (3). Data from the Annual Report on Drug Control in China indicated that the number of registered synthetic drug users exceeded users of traditional drugs such as heroin. At the end of 2016, the number of registered synthetic drugs users reached 1.52 million, accounting for 60.5% of all drug users (4–6). The use of MA contributes to a wide range of physical and mental health disorders.

It is well established that MA can induce both prolonged and transient psychosis, which is characterized typically by persecutory delusions and auditory hallucinations, but also delusions of reference, visual hallucinations, and thought broadcasting (7–9). MA-associated psychotic symptoms give rise to a variety of adverse consequences that are considerable public health concerns, including negatively impacting on the individual's quality of life and increasing the burden on their family and society (10, 11). Studies conducted internationally provide evidence that MA-associated psychotic symptoms increase risk for suicidality (10–12).

The increasing use of MA and associated harms globally raise questions regarding the prevalence of MA-associated psychotic symptoms in MA users. The reported prevalence of psychotic symptoms among MA users varies widely in different countries, ranging between 13 and 46% (9, 13–18). A Chinese study reported 17.4% prevalence of psychotic symptoms in MA users from detoxification centers (19). Studies have shown that earlier, longer and heavier use of MA was associated with increased risk for MA-associated psychotic symptoms (19–21). MA dependence, too, may increase the risk for MA-associated psychotic symptoms (9, 13–16). There is also an association between increased prevalence of co-occurring anxiety and depressive symptoms in individuals with MA-related psychotic symptoms (22, 23). Most studies to date have been conducted in developed countries, however, and evidence from developing country populations is scant and interpretation limited by small sample size. This study aimed to examine the prevalence of psychotic symptoms among MA users in China, and to systematically analyze the risk factors for MA-associated psychotic symptoms among Chinese MA users.

## Materials and methods

### Participants

A cross-sectional study of MA–associated psychotic symptoms and risk behavior among MA users was conducted in compulsory drug detoxification centers in Beijing and Guangdong Province, China. MA users who only or primarily used MA in the past year (prior to enrollment) were recruited from April, 2012 to October, 2015 by convenient sampling method. Inclusion criteria were: age 18 years or above; MA as primary drug ever used, and positive urine test for MA at time of entry to the treatment center. Subjects were excluded if they had significant physical illnesses, such as cerebrovascular disease, cardiovascular disease, or stroke which had been diagnosed by doctors previously. The participants who declined to participate or otherwise did not participate were not disadvantaged in any way. Written informed consent was obtained from all the study participants. The study was approved by the Institutional Ethics Board of Peking University. A total of 1685 MA users who reported that MA was their primary drug ever used and completed the psychotic symptoms assessment were included in the present study.

### Measures

#### Sociodemographic and drug use questionnaire

A self-designed structured questionnaire that collected information on demographic characteristics and drug use history was administered in face-to-face interviews by trained interviewers. Demographic variables included geographical area of survey, age, gender, ethnicity, employment, education, and marital status. Information about drug use included: synthetic drug ever used, other substance ever used, two or more synthetic drugs ever used, main route, source, cause, sites of MA administration, dose, frequency and duration of MA use, concurrent use of other drugs and MA dependence in the past year before entering the treatment center. MA dependence was defined as present if there were two or more of the following symptoms: craving, tolerance, withdrawal, out-of-control drug use, preoccupation with drug, and use despite significant impairment (based on the Diagnostic and Statistical Manual of Mental Disorders, 4th edition (24).

#### The positive and negative syndrome scale (PANSS)

The Positive and Negative Syndrome Scale (PANSS) was used to assess psychotic symptoms (25). The 30-item scale assesses the severity of positive syndrome (7 items), negative syndrome (7 items), and general psychopathology (16 items). Each item rated on a 7-point scale (1=absent, 2=minimal, 3=mild, 4=moderate, 5=moderate severe, 6=severe, and 7=extreme), with cumulative scores ranging from 30 to 210. Clinically significant psychotic symptoms corresponded to a PANSS total score of 58 and above (26). The validity and reliability of PANSS (Chinese version) both meet the requirements of psychometrics for assessing psychotic symptoms in Chinese patients, with internal consistency reliability of 0.87 (27).

#### The beck depression inventory (BDI)

Symptoms of depression were measured using the short 13-item Beck Depression Inventory (BDI). Each item is scored from 0 to 3 with total scores ranging from 0 to 39. A score of ≥8 was classified as indicative of having depressive symptoms (28). Previous research demonstrates that the BDI can be reliably used to assess depression symptoms in Chinese populations (29).

#### The hamilton anxiety scale (HAMA)

Anxiety symptoms were measured using the Hamilton Anxiety Scale (HAMA). The scale comprises 14 items each rated from 0 to 4, with cumulative scores ranging from 0 to 56. A cut-off value of ≥14 was used to define presence of anxiety symptoms (30). HAMA has been demonstrated to have good reliability and validity in Chinese individuals (31).

#### The barratt impulsive scale (BIS-11)

The Barratt Impulsive Scale (BIS-11) was used to measure impulsiveness and comprises 30 items (32). In the adapted Chinese version, each of the 30 items is scored from 1 to 5, with total scores ranging from 30 to 150 (33). The higher the total questionnaire score, the higher the individual's level of impulsiveness (34). Reliability and validity in Chinese populations are acceptable (33).

### Statistical analysis

EpiData software (Odense, Denmark) was used to verify consistency of the double-entered data. Descriptive analysis was conducted for the demographic and drug use characteristics, psychotic symptoms, depressive symptoms, and anxiety symptoms of the study participants with the estimated means and proportions. Univariate logistic regression was used to examine the association of variables with risk for MA-associated psychotic symptoms. Multiple logistic regression was conducted using a stepwise backward sequence. Significant variables from univariate analysis were included for multiple logistic regression analysis. All the statistical tests were completed using Stata version 13.1 (35). Statistical significance was set at 0.05 in two-sided tests for all analyses.

## Results

### Sample characteristics and pattern of drug use

1685 MA users participated in the study, from Beijing (43.2%) and Guangdong (56.9%) Province, China. Tables 1, 2 show the demographic and drug use characteristics of the subjects respectively. Age ranged from 18 to 57 years (mean, 32.9 years) and 655 participants (39.5%) were < 30 years old. The majority were male (86.4%), of Han ethnicity (92.8%), 52.0% were unemployed, and 48.6% unmarried. The level of education was mainly junior high school or below (78.4%).

**Table 1:** Characteristics of methamphetamine users.

**Variable**	**Samples (*n*)**	**Proportion (%)/ Mean ± SD**	**Variable**	**Samples (*n*)**	**Proportion (%)**
Area^*^			Employment status^*^		
Beijing	725	43.15	Unemployed	869	52.04
Guangdong	955	56.85	Employed	801	47.96
Age (years)^*^	1657	32.89 ± 8. 51	Education^*^		
<30	655	39.53	Primary school or below	388	23.43
≥30	1002	60.47	Junior high school	911	55.01
Gender^*^			Secondary technical school	88	5.31
Male	1420	86.37	Senior high school or above	269	16.24
Female	224	13.63	Marital status^*^		
Ethnicity^*^			Unmarried	799	48.57
Han	1511	92.81	Married/Cohabitating	597	36.29
Minority	117	7.19	Divorced, separated, widowed	249	15.14

* *With missing value*.

**Table 2:** Drug use characteristics and psychosis symptoms of the methamphetamine users.

**Variables**	**Samples (*n*)**	**Proportion (%)/Mean ± SD**
**SYNTHETIC DRUG EVER USED**^#^		
Methamphetamine	1685	100
Ketamine	83	4.94
Magu (Main component is MA)	63	3.75
Ecstasy	56	3.34
Marijuana	35	2.08
**OTHER SUBSTANCE EVER USED**^#^		
Heroin	237	14.25
Tobacco	565	33.97
Alcohol	312	18.76
TWO OR MORE SYNTHETIC DRUGS EVER USED^*^		
No	1486	88.51
Yes	193	11.49
**PRIMARY ROUTE OF MA ADMINISTRATION**^#^		
Smoking/insufflation	1586	95.48
Intravenous	31	1.87
Snorting	15	0.90
Oral	18	1.08
**SOURCE OF DRUG**^#^		
Illegal drug market	1222	73.17
Dance halls and other entertainment place	258	15.45
Friends / relatives	471	28.20
Buy online	30	1.80
**CAUSE OF MA USE**^#^		
Craving	864	57.71
Influence by friends or peers	757	45.30
Pursuit of euphoria	674	40.34
Anti-fatigue, “mention spirit”	494	29.56
Emotions, emotional problems	255	15.26
To socialize	103	6.16
Trying to “enhance sexual function”	67	4.01
Trying to “lose weight”	48	2.87
**DRUG USE SITES**^#^		
At home	834	49.82
Friend's house	603	36.02
Hotel / restaurant	516	30.82
Dance halls / disco	303	18.10
Bathing center	96	5.73
Age at first MA use (years)^*^	1636	28.48 ± 8.07
≤25	654	39.98
>25	982	60.02
**DOSE OF METHAMPHETAMINE USE**		
< 0.2 g per time	879	52.17
≥0.2 g per time	806	47.83
CONCURRENT USE OF OTHER DRUGS^*^		
No	1589	95.95
Yes	67	4.05
PREVIOUS DRUG TREATMENT^*^		
No	1325	79.96
Yes	332	20.04
FREQUENCY OF METHAMPHETAMINE USE^*^		
1 time/day	295	17.76
2–6 times/week	322	19.39
1 time/week	397	23.90
1–3 times/month	410	24.68
<1 times/month	237	14.27
**DURATION OF METHAMPHETAMINE USE (MONTHS)**		
≤3	466	27.66
>3	1219	72.34
METHAMPHETAMINE DEPENDENCE^*^		
No	578	35.20
Yes	1064	64.80
LENGTH OF ABSTINENCE^*^		
≤1 month	759	55.20
>1 month	616	44.80
PSYCHOTIC SYMPTOMS^**^		
Psychosis symptoms(PANSS ≥ 58)	286	16.97
Depressive symptoms (BDI ≥ 8)^*^	664	40.54
Anxiety symptoms (HAMA ≥ 14)^*^	485	29.06

# *Multiple choices*.

* *With missing value*.

** *PANSS, The Positive and Negative Syndrome Scale; BDI, Beck Depression Inventory; HAMA, Hamilton Anxiety Scale*.

Among the 1685 MA users, 14.3% had a history of heroin use, 18.8 and 34.0% reported alcohol use and cigarette smoking respectively. The majority (95.5%) used MA by smoking or insufflation. Craving (57.7%), influence by friends or peers (45.3%), and pursuit of euphoria (40.3%) were the main reasons given for drug use. The average age at onset of MA use was 28.5 years, 40.0% of the MA users' age at first MA use was ≤ 25 years. 72.3% had used MA for more than 3 months, 61.1% had used MA every week. 64.8% were MA-dependent. 55.2% of MA users were abstinent for 1 month or less. Almost half (47.8%) had used ≥0.2 g per time. 20.0% had received prior treatment for drug use.

### Prevalence and profiles of psychotic and affective symptoms

286 (17.0%) had MA-associated psychotic symptoms. Table 3 also shows the proportion to which each item psychotic symptom on the PANSS was rated as present among MA users who met criteria for psychosis (PANSS> = 58). The top three psychotic symptoms were somatic concern (G1, 36.7%), guilt feelings (G3, 36.7%), and excitement (P4, 33.9%), respectively. The other three noteworthy psychotic symptoms were hallucinations (P3, 27.3%), suspiciousness/persecution (P6, 26.6%), and delusions (P1, 17.8%).

**Table 3:** Prevalence of PANSS symptoms reported by methamphetamine users who met criteria for psychosis (PANSS > = 58).

Symptom^*^	***N* (for Item≥4)**	**Percent (%)**
**POSITIVE SCALE**		
Delusions(P1)	51	17.83
Conceptual disorganization(P2)	70	24.48
Hallucinations(P3)	78	27.27
Excitement(P4)	97	33.92
Grandiosity(P5)	59	20.63
Suspiciousness/persecution(P6)	76	26.57
Hostility(P7)	48	16.78
**NEGATIVE SCALE**		
Blunted affect(N1)	56	19.58
Emotional withdrawal(N2)	52	18.18
Poor rapport(N3)	64	22.38
Passive/apathetic social withdrawal (N4)	52	18.18
Difficulty in abstract thinking (N5)	49	17.13
Lack of spontaneity and flow of conversation(N6)	63	22.03
Stereotyped thinking (N7)	52	18.18
**GENERAL PSYCHOPATHOLOGY SCALE**		
Somatic concern (G1)	105	36.71
Anxiety(G2)	75	26.22
Guilt feelings(G3)	105	36.71
Tension (G4)	92	32.17
Mannerisms and posturing (G5)	45	15.73
Depression(G6)	83	29.02
Motor retardation (G7)	59	20.63
Uncooperativeness (G8)	49	17.13
Unusual thought content(G9)	55	19.23
Disorientation (G10)	44	15.38
Poor attention(G11)	56	19.58
Lack of judgment and insight (G12)	60	20.98
Disturbance of volition (G13)	59	20.63
Poor impulse control(G14)	68	23.78
Preoccupation (G15)	58	20.28
Active social avoidance (G16)	67	23.43

* *Score of item ≥ 4*.

### Predictors of MA-associated psychotic symptoms

The multiple logistic regression (Table 4) showed that a higher dose (≥0.2 g per time), and a longer duration of MA use (>3 months) were both associated with increased odds of psychotic symptoms: adjusted odds ratio (OR) = 1.96 (95% confidence interval [CI]: 1.40–2.76), and 1.98 (95% CI: 1.33–2.96), respectively. Subjects who reported a history of heroin or tobacco use had higher odds of MA—associated psychotic symptoms than those without (OR = 2.45, 95% CI: 1.67–3.60; OR = 1.78, 95% CI: 1.27–2.49, respectively). Being married/cohabitating, or unemployed had lower odds of MA-associated psychotic symptoms: OR = 0.56 (95% CI: 0.39–0.81) and 0.65 (95% CI: 0.47–0.92), respectively. MA users with anxiety symptoms or depression symptoms had significantly increased odds of having MA-associated psychotic symptoms by 9.70 (95% CI: 6.92–13.59) and 1.90 (95% CI: 1.36–2.65) times. Based on the average score of BIS-11 (75.79 ± 21.24), we divided the subjects into high impulsivity group (BIS-11>75) and low impulsivity group (BIS-11≤75). Individuals with high impulsivity were more likely to have MA-associated psychotic symptoms than those with low impulsivity: OR = 2.19 (95% CI: 1.50–3.20).

**Table 4:** Logistic regression analysis of the risk factors for psychotic symptoms (PANSS total score ≥ 58) in methamphetamine users.

**Variables**	**Samples(*n*)**	**Psychotic symptoms (%)**	OR(95%CI)^*^	**AOR(95%CI)^#^**
**SEX**				
Female	224	13.84	1.00	
Male	1420	16.97	1.27 (0.85–1.91)	
**AGE (YEARS)**				
<30	655	17.71	1.00	
≥30	1002	16.57	0.92 (0.71–1.20)	
**ETHNICITY**				
Han	1511	16.68	1.00	
Minority	117	18.80	1.16 (0.71–1.88)	
**EDUCATION**				
Primary school or below	388	18.04	1.00	
Junior high school	911	16.90	0.92 (0.68–1.26)	
Secondary technical school	88	21.59	1.25 (0.71–2.21)	
Senior high school or above	269	14.50	0.77(0.50–1.18)	
**MARITAL STATUS**				
Unmarried	799	18.40	1.00	1.00
Married/Cohabitating	579	14.24	0.74 (0.55–0.99)	0.56 (0.39–0.81)^**^
Divorced, separated, widowed	249	18.47	1.01 (0.70–1.45)	0.81 (0.51–1.30)
**EMPLOYMENT STATUS**				
Employed	801	22.22	1.00	1.00
Unemployed	869	12.08	0.48 (0.37–0.63)	0.65 (0.47–0.92)^**^
**AGE AT FIRST MA USE**				
≤25	117	17.89	1.00	
>25	161	16.40	0.90 (0.69–1.17)	
**DOSE OF METHAMPHETAMINE USE**				
< 0.2 g per time	879	10.24	1.00	1.00
≥0.2 g per time	806	24.32	2.82 (2.15–3.69)	1.96 (1.40–2.76)^**^
**TWO OR MORE SYNTHETIC DRUGS EVER USED**				
No	1486	14.87	1.00	
Yes	193	33.68	2.91 (2.09–4.05)	
**PREVIOUS DRUG TREATMENT**				
No	1325	17.28	1.00	
Yes	332	16.87	0.97 (0.71–1.34)	
**WEEKLY METHAMPHETAMINE USE**				
No	647	13.45	1.00	
Yes	1014	19.13	1.52 (1.16–2.00)	
**DURATION OF METHAMPHETAMINE USE (MONTHS)**				
≤3	466	12.02	1.00	1.00
>3	1219	18.87	1.70 (1.24–2.33)	1.98 (1.33–2.96)^**^
CONCURRENT USE OF OTHER DRUGS^*^				
No	1589	15.48	1.00	
Yes	67	46.27	4.70(2.85-7.74)	
**METHAMPHETAMINE DEPENDENCE**				
No	578	11.94	1.00	
Yes	1064	18.98	1.73(1.29-2.32)	
**LENGTH OF ABSTINENCE**				
≤1 month	129	17.00	1.00	
>1 month	107	17.37	1.03 (0.77–1.36)	
**HISTORY OF HEROIN USE**				
No	1426	13.74	1.00	1.00
Yes	237	37.13	3.71 (2.74–5.02)	2.45 (1.67–3.60)^**^
**HISTORY OF TOBACCO USE**				
No	1098	14.39	1.00	1.00
Yes	565	22.30	1.71 (1.32–2.22)	1.78 (1.27–2.49)^**^
**HISTORY OF ALCOHOL USE**				
No	1351	17.32	1.00	
Yes	312	16.03	0.91 (0.65–1.27)	
**ANXIETY SYMPTOMS**				
No	1194	6.00	1.00	1.00
Yes	485	43.71	12.17 (9.02–16.43)	9.70 (6.92–13.59)^**^
**DEPRESSION SYMPTOMS**				
No	974	11.29	1.00	1.00
Yes	664	24.25	2.51 (1.93–3.28)	1.90 (1.36–2.65)^**^
**BARRATT IMPULSIVE SCALE**				
≤75	636	8.65	1.00	1.00
>75	1049	22.02	2.98 (2.18–4.08)	2.19 (1.50–3.20)^**^

* *Univariate logistic regression analysis*.

# *Multiple logistic regression analysis. Adjusted effects including only those factors that showed significant effects in the univariate analysis, empty cells indicate variables not included in the final model conducted using a stepwise backward sequence*.

** *There was significant difference, p < 0.05*.

## Discussion

The present study found that MA-associated psychotic symptoms were common (17%) among abstinent MA users in detention centers in Beijing and Guangdong Province, China. Factors that increased risk for MA-associated psychotic symptoms included both longer duration and a higher typical dose of MA use. Other factors included a history of heroin or tobacco use, comorbid depression or anxiety symptoms, and higher impulsivity. Though this study cannot demonstrate a causal link, important next steps include examination of the effects of comprehensive interventions to assist in reducing the use of MA, actively treating depression and anxiety comorbidity, and relieving impulsivity through psychological and behavioral interventions in reducing the risk for MA-associated psychotic symptoms in this population.

The prevalence of MA-associated psychotic symptoms reported here is similar to the rate reported in an Australian study, which excluded MA users with pre-morbid psychotic symptoms (17.0% vs. 18%) (17). Prior research showed that the risk of experiencing psychotic symptoms in MA users who were abstinent ranged from 12.7 to 26.4% (21). The nature of psychotic symptoms has been remarkably consistent across previous studies, with high frequency of hallucinations, delusions of persecution, thought broadcasting, and suicidal ideation (7, 9, 20, 21).

Regular use of a higher dose of MA was associated with higher risk for MA-associated psychotic symptoms, consistent with earlier studies that indicated that heavier MA users are at higher risk of psychosis (17, 36–38). Similarly, MA users with a longer duration of drug use were more likely to have MA-associated psychotic symptoms, concordant with findings from a longitudinal prospective cohort study that showed a strong dose-response effect between number of days of MA use and psychotic symptoms (20). A similar of a dose-response relationship between the duration of MA use and risk of psychiatric symptoms was observed in our previous study (19). Higher dose and longer duration of MA use represent greater severity of exposure to MA. These findings suggest that reducing MA use may be helpful to reduce the risk of psychosis among this population. Our study found that history of heroin and tobacco use each is associated with higher risk for occurring MA-associated psychotic symptoms. Although heroin use is rarely reported as a risk factor in MA-associated psychotic symptoms, previous research has demonstrated that mental health among heroin users is poorer than in the general population (39). Smoking as a risk factor for MA-associated psychotic symptoms has been reported by a Chinese study conducted in a Compulsory Drug Detoxification Center (19).

Being of married or cohabitating status was associated with reduced risk for psychotic symptoms, findings which concur with our previous study. It may be that being in a longterm relationship is a protective factor against MA-associated psychotic symptoms. Alternatively, it may be that those who can sustain a relationship have different characteristics that render them less likely to be vulnerable to the development of psychotic symptoms. Being unemployed was also a protective factor but this finding was contrary to previous research results (40). Future study is necessary to further examine this issue. High prevalence of anxiety symptoms and depressive symptoms were reported in the study, and users with comorbid anxiety symptoms or depressive symptoms were more likely to experience MA-associated psychotic symptoms than users without, consistent with previous studies (19, 38).

MA users with high impulsivity were more likely to experience MA-associated psychotic symptoms than those with low impulsivity. Impulsivity has been shown previously to be associated with earlier initiation of substance use and greater MA consumption (41, 42). MA use itself is associated with heightened impulsivity in animal models (43). Impulsivity is a multidimensional construct, and studies have shown that impulsivity may be related to damage to dopamine and serotonin axons, dopamine D2/D3 receptor and polymorphism of *COMT* (Catechol-O- methyltransferase), *DAT* (Dopamine transporter), and *DRD4* (Dopamine D4 receptor) genes (44–46), which may be implicated in the risk for MA-associated psychotic symptoms (9, 47–50). Further research of the relationship between MA-associated psychotic symptoms and impulsivity is needed. The association with impulsivity is important when considering interventions aimed at the prevention and treatment of MA-associated psychotic symptoms: presence of impulsivity is likely to indicate additional risk for psychosis, but its presence is likely to be associated with poorer adherence to treatment and irregular engagement and help-seeking.

Previous studies have reported that psychotic symptoms largely resolved within a week of cessation of MA among MA-dependent participants (51), but the data from this study did not support those findings. The reason may be that the participants of this study were recruited from the Compulsory Drug Detoxification Center where all individuals entered after staying in detention centers for a few days and thus had longer abstinence periods than participants recruited in other study designs. We did not find a statistically significant association between the age of onset of MA use and MA-associated psychotic symptoms. Evidence on onset of MA use and risk for psychotic symptoms varies across studies, and further research is required (52, 53). Interestingly, the symptoms and some risk factors of MA-associated psychotic symptoms are similar to those observed in cocaine-induced psychosis (54, 55). Both MA and cocaine belong to the psychostimulants class, and are highly related, so the findings in this study possibly could be extrapolated to other psychostimulant-related psychoses.

This study included a large sample that was drawn from compulsory drug detoxification centers in Beijing and Guangdong Province, China. The extent to which the findings reported here are generalizable to users of MA within the community is unclear. The present study relied on retrospective self-reports of drug use history. This may have resulted in recall bias. The assessment of psychiatric symptoms was not based on DSM-based structured interviews, which may have affected the accuracy of the prevalence of psychotic symptoms. We did not collect information on psychiatric histories, particularly past history of psychotic illness, which may be an important confounding factor in the relationship between MA use and psychotic symptoms (17, 56). In addition, other potentially relevant risk factors such as family history of psychosis or psychiatric illness, personality traits, and adverse childhood experiences were not assessed in this study. In spite of these limitations, we reported the prevalence of and risk factors for MA-associated psychotic symptoms among MA users in a large population in China. The finding that higher impulsivity is associated with greater risk for psychotic symptoms is novel and warrants further research. Given the widespread use of methamphetamine in China in recent years, greater awareness is needed about the potential effect of this drug on mental health issues. There is a need to provide integrated care for patients who suffer from substance use disorders and psychiatric disorders to enable better prevention and treatment.

## Conclusion

This study found that MA-associated psychotic symptoms were common in MA users, and identified that higher dose and longer duration of MA use, history of heroin or tobacco use, depression or anxiety symptoms, and higher impulsivity were each associated with higher risk for psychotic symptoms. These findings highlight the need to develop prevention and treatment strategies for psychiatric symptoms, and psychotic symptoms in particular, among MA users.

## Author contributions

LL and YB designed the study and oversaw data collection. M-FS and M-XL designed the data analytic plan, performed literature review, and wrote the manuscript and tables. J-QL conducted analyses and made contributions to the content of the manuscript. JL, SL, PW, Z-ML and JS revised the manuscript and approve the final manuscript.

### Conflict of interest statement

The authors declare that the research was conducted in the absence of any commercial or financial relationships that could be construed as a potential conflict of interest.
